# Effects of intermittent hypoxia-hyperoxia on mobility and perceived health in geriatric patients performing a multimodal training intervention: a randomized controlled trial

**DOI:** 10.1186/s12877-019-1184-1

**Published:** 2019-06-14

**Authors:** Ulrike Bayer, Rudolf Likar, Georg Pinter, Haro Stettner, Susanne Demschar, Brigitte Trummer, Stefan Neuwersch, Oleg Glazachev, Martin Burtscher

**Affiliations:** 1 Department of Geriatrics, Klinikum Klagenfurt, Kraßniggstraße 2, 9020 Klagenfurt am Wörthersee, Austria; 2 Department of Anesthesiology and Intensive Care Medicine, Klinikum Klagenfurt, Feschnigstr. 11, 9020 Klagenfurt, Austria; 3 Department of Statistics, Alpen-Adria University Klagenfurt, Universitätsstraße 65-67, 9020 Klagenfurt, Austria; 4 Science and Technology Park for Biomedicine, I.M, Sechenov First Moscow State Medical University, Trubetskaya Str. 8-2, 119991 Moscow, Russia; 5 Department of Sport Science, Medical Section, University of Innsbruck, Fürstenweg 185, 6020 Innsbruck, Austria

**Keywords:** Elderly, Multimodal training, Hypoxia, Intervention, Perceived health

## Abstract

**Background:**

Additional benefits of passive exposures to intermittent hypoxia and hyperoxia on cognitive performance and functional exercise capacity have been demonstrated in geriatric patients who performed a multimodal training program. The main goal of the present study was to evaluate effects of adding intermittent hypoxic-hyperoxic training (IHHT) to a multimodal training intervention (MTI) on mobility and perceived health in old individuals at a Geriatric Day Hospital.

**Methods:**

Thirty-four patients between 64 and 92 years participated in the double blind, randomized and controlled clinical trial. The elderly patients attended in a 5–7 weeks lasting MTI (strength, endurance, balance, reaction, flexibility, coordination, and cognitive exercises) and performed IHHT (breathing 10–14% oxygen for 4–7 min followed by 2–4 min 30–40% oxygen) in the Hypoxic Group (HG) or placebo treatment with ambient air in the Normoxic Group (NG) in parallel. Before and after all treatments, mobility was assessed by the Tinetti Mobility Test (TMT), the Timed-Up-and-Go Test (TUG) and Barthel-Index, while perceived health was assessed by one part of the EQ-5D Test, the EQ visual analogue scale (EQ VAS).

**Results:**

After the MTI plus IHHT or normoxia sessions, results of the TMT, TUG, Barthel Index and EQ-VAS revealed no significant difference between HG and NG (+ 14.9% vs + 15.4%, *p* = 0.25; − 21% vs − 26.3%, *p* = 0.51; + 4.2% vs + 3.6%, *p* = 0.56; + 37.9% vs + 33.9%, *p* = 0.24;).

**Conclusions:**

IHHT added to MTI did not elicit additional improvements in perceived health and mobility compared to MTI alone.

## Background

Worldwide, life expectancy at birth rose by 6.2 years from 65.3 years in 1990 to 71.5 years in 2013 [1]. Although the life expectancy in good health increases too, its extent is lower by far, suggesting that the world’s population currently loses more years of healthy life due to disability than 20 years ago [2]. Therefore, the Quality of Life (QoL) in old age is decreasing despite the fact that medical care is improving worldwide. Physical disability is the main reason for low QoL; elderly people want to be independent to feel comfortable, as reported by Jalavondeia et al. [3]. Also, Langlois et al. [4] showed in their study that physical exercise improves not only physical capacity but also the QoL. With advancing age, inactivity increases, gait and balance change and the increase in chronic diseases with high prescription medication use leads to a higher risk of falls [5]. Falls not only reduce the QoL, the treatment of fall injuries is very costly. Stevens et al. estimated the total direct medical costs for falls including what patients and insurance companies paid $34 billion for in the United States of America alone in 2013. With a constantly increasing population, the number of falls and the costs to treat fall injuries are likely to rise [6]. There are many different risk factors such as disability of the lower extremities, abnormalities of balance and gait, foot problems and cognitive impairment; the risk of falling increases linearly with the number of risk factors [7]. Tinetti et al. showed that a multimodal individual training intervention including physical training, balance and strength training not only reduced the number of risk factors and led to a reduction in the incidence and to an improvement in functional independence, but also increased confidence among elderly patients in performing their daily activities [8]. However, it is not only physical functioning but also bodily pain, social functioning, mental health, and emotional problems which affect the QoL [9].

The Geriatric Day Hospital in Klagenfurt (Carinthia, Austria) is one of the institutions that provides a multimodal training intervention (MTI), which is specially tailored for geriatric patients to improve mobility, cognitive function, mental health, and QoL. In addition to this physical training intervention, and due to the low resilience of geriatric patients, we were also looking for new strategies and found that Intermittent Hypoxic-Hyperoxic Training (IHHT) not only led to an additional increase in exercise performance but also to an increase in cognitive function and a decrease in pain [10]. However, psychological factors, risk of falling and short-term mobility were not considered.

We suspected that that IHHT might be a promising treatment in addition to a MTI regarding mobility and QoL. In comparison to known effects of Intermittent Hypoxic Training (IHT) [11–15], IHHT may cause more beneficial effects. Hyperoxic periods with 30–40% oxygen, compared to normoxic breathing, result in a faster recovery of oxygen desaturation after hypoxic periods [16]. IHHT was suggested to produce a faster membrane-stabilizing effect in cells of the heart, liver and brain compared to IHT in a study with male Wistar rats [17]. This new approach is more economical than IHT, as the recovery time between bouts of hypoxia exposure is shortened to 3 min, allowing for a higher number of hypoxia–hyperoxia cycles during the same session [18]. Exercise combined with hypoxic training also improved cognitive function of older individuals [14], and the addition of hyperoxic intervals might have accelerated clearance of metabolites negatively impacting on neuronal metabolism in dementia [19]. On the other hand, however, it was shown that metabolites like lactate have a positive effect on the brain metabolism [20]. IHHT is well-tolerable and applicable to geriatric patients without any negative side effects [10] and has been shown to even improve exercise tolerance and aerobic capacity in patients without any additional exercises [21]. As it was demonstrated that intermittent hypoxia improved QoL in elderly subjects [14] and enhanced walking after chronic spinal cord injury [22], we seek to evaluate IHHT effects on mobility and perceived health, which is an important part of the complex construct of QoL in geriatric patients.

We hypothesised that IHHT combined with MTI would more favourably impact on mobility and perceived health than MTI alone. Thus, the aim of the present study was to explore the effects of IHHT added to MTI on mobility and perceived health in geriatric patients.

## Methods

### Participants and randomisation

**Fig. 1:**
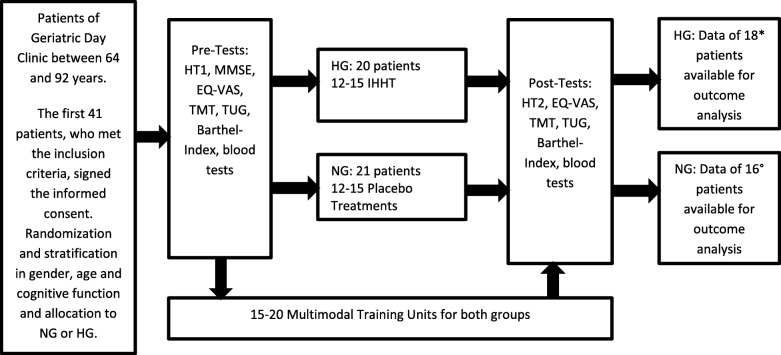
Process of inclusion, randomization, stratification, training program and outcome analysis

Forty-one geriatric patients between 64 and 92 years participated in this stratified, randomised and double-blind study. The study was performed at the Geriatric Day Clinic in Klagenfurt (Carinthia, Austria), this is a semi-stationary facility in the house of geriatrics offering elderly people the possibility of rehabilitation with the goal: to enable the elderly to live as independently as possible in health at home. All patients of the Geriatric Day Clinic suffer from several different diseases. The most frequent diagnoses of the study participants were arterial hypertension, condition after surgery of a total endoprosthesis, partially after fractures due to falls, osteoporosis, degenerative spinal diseases, arthrosis, dementia development, atrial fibrillation, heart failure, coronary heart disease, diabetes mellitus, renal insufficiency, gastrointestinal diseases and depression. The patients were randomly assigned to the hypoxic group (HG) and the normoxic group (NG). The process of inclusion, randomization, stratification, training program and outcome analysis is presented in Fig. 1, the baseline characteristics in Table 1. A more detailed description of the participants and methods is provided in our previous paper [10]. All comorbidities, therapies, and interventions of medical care were documented in written documents and also electronically stored in the intranet of the hospital.

**Table 1:** Baseline characteristics of all patients with no difference between groups

	Hypoxic group (*n* = 18)	Normoxic group (*n* = 16)	*p*-value
Gender (m, f)	m 5 (28%) / f 13 (72%)	m 2 (12.5%) / f 14 (87.5%)	0.25^b^
Age (years)	80.89 (7.87)	83.44 (5.5)	0.14
Height (cm)	163.72 (8.29)	163.19 (8.52)	0.43
Weight (kg)	72.03 (9.32)	66.83 (12.27)	0.09
BMI (kg/m^2^)	26.98 (3.91)	25.02 (3.62)	0.07
MMSE score	24.94 (3.75)	24.5 (3.93)	0.36
Therapy days (n)	18.33 (2.43)	17.5 (2.58)	0.16^c^
Arterial oxygen saturation (%)	94.21 (6.19)	93.65 (7.54)	0.42
Minimum oxygen saturation (%)^a^	81.39 (2.8)	83.85 (5.51)	0.27^c^
Regular medication, n (%)			
Anticoagulants	9 (50%)	10 (62.5%)	0.35^b^
ß-blockers	7 (38.9%)	8 (50%)	0.38^b^
ACE inhibitors	7 (38.9%)	6 (37.5%)	0.61^b^
AT II inhibitors	6 (33.3%)	3 (18.8%)	0.29^b^
Calcium channel blockers	5 (27.8%)	1 (6.3%)	0.12^b^
Statins	1 (5.6%)	4 (25%)	0.13^b^
Diuretics	8 (44.4%)	9 (56.3%)	0.37^b^
Nitrates	1 (5.6%)	1 (6.3%)	0.73^b^

Data represent means (SD) or frequencies (%); there are no significant differences between the groups, *p* < 0.05-Exact Fisher Yates Test; MMSE: Mini-Mental State Examination. ^a^Minimum arterial oxygen saturation measured in the Hypoxic Test

For the measured values of each of the listed variables, the two difference lists (end value - initial value for normoxia, hypoxia) were tested for normal distribution using the Kolmogoroff-Smirnov test

*p*-values between the groups. If both lists were significantly normally distributed (*p* < 0.95), the *p*-value was determined by the t-test, otherwise by the (nonparametric) U-test marked with ^c^; comparison of rates is tested with Exact Fisher Test marked with^b^

At the end, data from 34 patients who successfully completed the entire study program were available for the outcome analysis (Fig. 1).

The study was approved by the local Ethics Committee (EK-Nr.: A 09/14) and performed in accordance with the ethical standards of the Declaration of Helsinki in 1975.

### Study protocol

#### Multimodal training intervention (MTI)

The intervention program was started after randomization, stratification, allocation to the HG or NG and Pre-Tests (Fig. 1). All 41 study participants came to the Geriatric Day Clinic in the morning 2–3 times a week and went home in the afternoon over a period of 5–7 weeks; this included 15–20 days of therapy, depending on the needs of each patient, using an individual treatment plan, which was documented in the medical recordings of the hospital. There was no difference between the groups with regard to therapy days, age, weight, height, gender and regular medication (Table 1). Both groups (NG and HG) attended the same individual rehabilitative care program, which was carried out and coordinated by a multi-disciplinary team of geriatricians, nurses, physiotherapists, and occupational therapists. The major goal of these therapies is to improve mobility, reduce the risk of falls and enable people to live as independently as possible at home. Therefore, the Geriatric Day Clinic provides an MTI with three focal points. The daily 30-min program of physiotherapy is based on strength training and functional exercises of the lower extremities and a combination of balance and reaction training, in order to enable the patients to walk as safely and as far as possible; a more detailed description can be found in our previous article [10].

#### Intermittent hypoxic–hyperoxic training (IHHT) programme

In parallel with the MTI, all patients underwent a Hypoxic Treatment with the ReOxy Breathing Therapy Device (AI Mediq S.A., Luxembourg). The device delivers a gas mixture with alternating oxygen content (10–30%) in nitrogen. Arterial oxygen saturation (SpO_2_) and pulse rate are measured continuously and stored. After nurses performed blood pressure measurements, patients from both groups (HG and NG) took part in the same 10 min lasting Hypoxic Test (HT) breathing a hypoxic gas mixture with 12% oxygen through a face mask while sitting in an armchair. After that, the device was able to establish an individually tailored IHHT for all subjects. Afterwards, patients of the HG repeatedly breathed hypoxic gas mixtures with 10–14% oxygen content lasting for 4–7 min depending on the individual reaction of the patients, followed by a 2–4 min exposure to a hyperoxic gas mixture with 30–40% oxygen content. During the hypoxic treatments, SpO_2_ and pulse rate were constantly monitored and transmitted to a monitoring device, which was invisible to the patients. The device compares the latest value of SpO_2_ with the predefined value of the individual’s minimum SpO_2_. As soon as a patient reaches the minimum SpO_2_ (Table 1), the device supplies immediately the hyperoxic gas mixture [23]. Also after the treatment, blood pressure was measured by nurses. The sessions of both groups lasted between 30 and 40 min with no visible difference for anyone except the study nurses, who provided the therapy and operated the devices.

The NG underwent the same HT but were only breathing a normoxic gas mixture during the treatments.

In total, 12–15 hypoxic or normoxic treatment procedures were performed for both groups 2–3 times a week over a period of 5–7 weeks, always together with the MTI on the same day (Fig. 1).

#### Assessments

The EQ-5D Test for Quality of Life and the Tinetti Mobility Test (TMT), the Timed-Up-and-Go Test (TUG) and the Barthel Index for mobility and fall risk were held out at the beginning before the hypoxic-hyperoxic and the normoxic sessions started and at the end after the last hypoxic-hyperoxic or normoxic treatment. We used these tests because they are part of the basic geriatric assessment in the Geriatric Day Clinic, are easy applicable for geriatric patients, are valid and reliable and all therapists are used to working with them. Fasting blood samples were also taken at the beginning and end of the study. We used the results of the Six-Minute Walk Test (6MWT), Clock-drawing Test (CDT) and Dementia Detection Test (DemTect) to establish whether improvements in exercise tolerance and cognitive function are related to perceived health in these patients. They were held out at the beginning and at the end like the other tests mentioned above, the exact results of all these tests can be found in our previous article [10].

#### Perceived health as a part of quality of life

We only used one part of the EQ-5D Test, the EQ visual analogue scale (EQ VAS), because the other part of this test is in our clinical experience too little meaningful. The EQ VAS self-rating records the respondent’s own assessment of health status [24], and it is in our daily experience with geriatric patients diagnostically conclusive. This provides us the opportunity to make a statement about the perceived health, an important part of QoL of the study participants. It provides a single index value for health status, takes only a few minutes and is cognitively simple; in our opinion, therefore, it is ideally suited for geriatric patients, even with cognitive impairment. It consists of a vertical, visual analogue scale from 0 to 100 similar to a thermometer, where 100 denotes the “Best imaginable health state” and 0 stands for the “Worst imaginable health state” [25]. The patients were asked to draw a line from the box on the left side to a number on the scale on the right side of the paper to indicate how their health is today. This value was taken for outcome analysis.

#### Mobility

The TMT is claimed to be the best predictor of fall risk and provides a dynamic assessment of mobility [26]. It is a simple and easily administered test and measures the patients’ gait and balance. The individual score of each patient is the combination of three measures: the overall gait assessment score, the overall balance assessment score and the gait and balance score. The total score is 28; a score between 19 and 24 indicates a risk of a fall, and a score below 19 shows a high risk of a fall [27].

The TUG is a reliable and valid test used to quantify functional mobility and predicts the patient’s ability to go outside alone safely [28]. The patient gets up from an arm chair and walks 3 m. Then, he turns and walks back to the chair and sits down again. The time required for the test is used for the outcome analysis, the shorter the time, the better the result.

The Barthel Index is a scale with a score of 0–100, which measures the performance in activities of daily life. Ten items describing ADL and mobility have to be performed by each patient. The less time and physical assistance that is needed, the higher the score is [29].

#### Cognitive testing

The Dem-Tect is a highly sensitive screening instrument to identify patients with MCI and patients with dementia in early stages and claimed to be more reliable than screening by the MMSE [30]. The CDT is also a valid and reliable screening test for dementia and cognitive impairment [31]. In our study we used the free-drawn method.

#### Evaluation of functional exercise capacity

The 6MWT was used to assess the functional exercise capacity of the study participants. It was carried out according to the *Guidelines for the 6-Minute Walk Test* of the American Thoracic Society [32].

### Statistical methods

Data are presented as means ± standard deviation (SD) or proportions. Unpaired t-tests (normally distributed data) and the Wilcoxon-Mann-Whitney U-test (not normally distributed data) were used to compare baseline data between groups as well as different changes (delta pre-post) between groups. Fisher exact test was used to compare proportions. Pearson or Spearman correlation analysis was performed to test relationships as demonstrated in 3.2.

A *p*-value < 0.05 was considered statistically significant.

## Results

The MTI did not cause any problems for elderly individuals. None of the patients were injured, although mild upper respiratory tract infections occurred in rare cases; however, all patients were able to complete the planned therapies. Also, the hypoxic-hyperoxic and normoxic sessions were well tolerated. There were no adverse side effects; in rare cases, sleepiness and slight dizziness were reported during hypoxic treatments. Overall, 34 of the 41 included patients successfully completed the entire study program, as shown in Fig. 1. Test results before and after treatments are shown in Table 2.

**Table 2:** Test results and differences of test results between HG and NG before and after treatments

	Hypoxic group (*n* = 18)	Normoxic group (*n* = 16)	Hypoxic group (*n* = 18)	Normoxic group (*n* = 16)	*p*-value
	Pre Post	Pre Post	Delta Pre-Post	Delta Pre-Post	between groups
EQ-VAS (0–100)	56.11 (11.95) 77.39 (11.1)	52.38 (13.48) 70.13 (8.88)	+ 21.28 (+ 37.9%)	+ 17.75 (+ 33.9%)	0.24
TMT (0–28)	17.56 (6.08) 20.17 (4.88)	18.19 (4.59) 21 (4.29)	+ 2.61 (+ 14.9%)	+ 2.81 (+ 15.4%)	0.25
TUG (seconds)	21.13 (8.62) 17.71 (6.86)	24.14 (17.05) 17,79 (6,08)	−4.44 (−21%)	−6.35 (−26.3%)	0.51
Barthel Index (0–100)	86.94 (15.06) 90.56 (9.84)	86.56 (11.21) 89.69 (8.65)	+ 3.61 (+ 4.2%)	+ 3.13 (+ 3.6%)	0.56

Data are means (SD); *Abbreviations*: *EQ VAS* visual analogue scale of the EQ-5D Test, *TMT* Tinetti Mobility Test, *TUG* Timed-Up-and-Go-Test (2 missing values in NG); normal distribution was tested with Kolmogoroff-Smirnov test; *p*-value: the significance was calculated with the t-test

### Mobility

Mobility was measured by the values of the TMT and the Barthel-Index and by the time needed for the TUG. Before and after the intervention, there were no significant differences between groups. After the intervention there was an improvement in all test results, but again with no difference between the two groups. IHHT did not lead to a significant additional improvement.

### Perceived health as a part of quality of life

Testing the perceived health with the EQ-5D revealed no significant difference between the groups at the beginning. The increase in the level of perceived health was somewhat higher in the HG but did not reach statistical significance compared to the NG (Table 2).

**Fig. 2:**
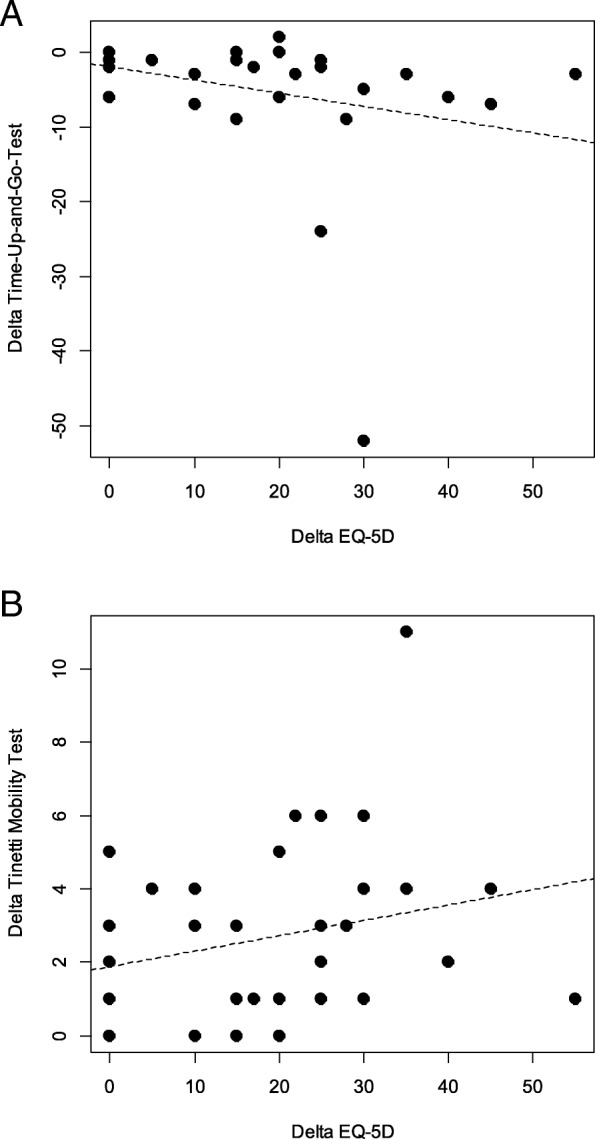
Correlations between the changes between (**a**) the Timed-Up-And-Go-Test (TUG) and the EQ visual analogue scale (EQ-5D) and (**b**) in the changes between Tinetti Mobility Test (TMT) and the EQ visual analogue scale (EQ-5D). **a** *r* = − 0.36, *p* = 0.02 (**b**) *r* = 0.27, *p* = 0.057

**Fig. 3:**
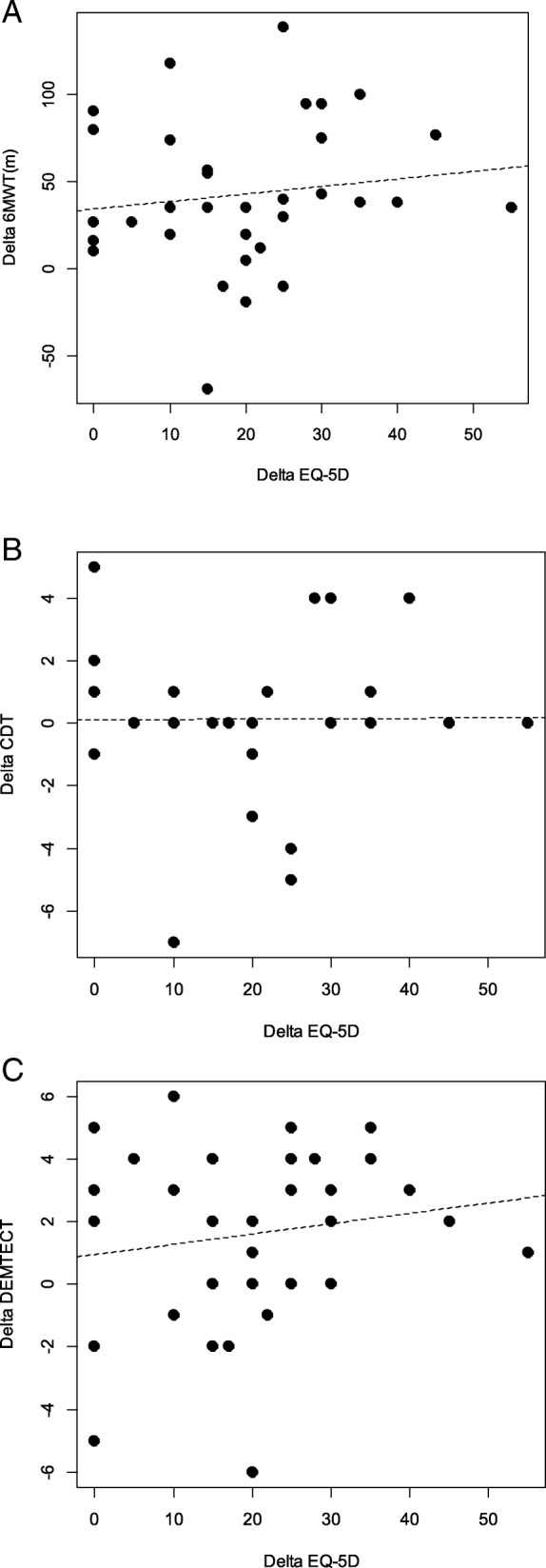
Correlations between the changes between (**a**) the Six-Minute-Walk-Test (6MWT) and the EQ visual analogue scale (EQ-5D) and (**b**) in the changes between Clock-Drawing-Test (CDT) and the EQ visual analogue scale (EQ-5D) and (**c**) in the changes between Dementia Detection Test (Dem-Tect) and the EQ visual analogue scale (EQ-5D). **a** *r* = − 0.23, *p* = 0.09 (**b**) *r* = − 0.048, *p* = 0.4 (**c**) *r* = 0.12, *p* = 0.24

Improvements in mobility and the decreased fall risk within the overall group were significantly correlated with an improvement in the EQ VAS, as shown in Fig. 2a and b. There was a significant negative correlation between the differences of the TUG and the EQ VAS (Fig. 2a) and a correlation between the differences of the TMT and the EQ VAS (Fig. 2b), which just failed to be statistically significant, with a *p*-value of 0.057, but showed a clear trend. Even the outliers do not influence these results. There was no significant correlation between EQ VAS and the Six-Minute-Walk-Test (*r* = − 0.23, *p* = 0.09), between EQ VAS and the Clock-Drawing-Test (*r* = − 0.048, *p* = 0.4) and between EQ VAS and the Dementia Detection Test (*r* = 0.12, *p* = 0.24) as shown in Fig. 3. A more detailed prescription of these test results can be found in our previous article [10].

### Blood tests and cardiorespiratory parameters

A slight but not significant decrease in systolic blood pressure, triglycerides and erythrocytes was observed within groups while the decrease in diastolic blood pressure was statistically significant within both groups. A significant decrease in resting heart rates was only found within the NG and a significant decrease in total cholesterol, HDL and LDL levels was observed in the HG. Arterial oxygen saturation and TNF alpha increased significantly after the intervention also only within the HG. None of these parameters changed differently between groups [10].

## Discussion

The aim of the present study was to explore potential benefits on mobility and perceived health, an important part of QoL in elderly patients when IHHT was added to MTI.

### Mobility

The risk of falling is especially high at values ​​below 19 in the TMT, as reported by Tinetti [26] and the “Kompetenz-Zentrum-Geriatrie” in Germany report [33]. This is also true for both groups before intervention, with a mean value below 19. After the intervention, both groups revealed a mean value greater than 19, which indicates a change from a high to a medium risk for falls [26]. A TMT score of 11 or less is even predictive of day hospital patients having a history of recurrent falls [34]*.* The TMT tests predictive and reactive balance mechanisms [35]; as so many body systems are needed to maintain balance [36], Thomas et al. recommend the TMT as a potential screening tool to identify patients at risk for falls in their pilot study. The scores of the TMT showed significant differences between fallers and non-fallers in this retrospective study [34]. Also, the TUG is claimed to predict falls, as other studies have reported, the shorter the time the better the result [37, 38]. A TUG with > 12 s is one of the most evidence-supported functional measures to determine individual risk of future falls, as reported by Delbaere et al. [39]. This reveals that our study participants, with a mean TUG of more than 12 s in both groups before intervention, have a high risk of falls. The TUG has almost as high a sensitivity and specificity as the TMT and contains components such as sit-to-stand, gait and turning, which are important aspects of postural control and are functionally important because many falls occur while walking [34]. The causes of falls are diverse and complex, but IHHT, which has positive effects on memory and exercise tolerance [10, 21], seems not to be an appropriate therapy for fall reduction. We were not able to confirm the hypothesis that IHHT would significantly contribute to expected improvements in the MTI. This study demonstrates improved mobility in both groups through MTI. However, since we did not include a control group, no valid statement can be made.

### Perceived health as a part of QoL

In our study, IHHT did not improve perceived health assessed with the use of EQ VAS. Various factors influence the subjective perception of quality of life. Perceived social support and marital status are associated with improved psychological health and QoL [40], as well as a higher socio-economic position [41]. All of these factors can hardly be influenced by therapy, but the geriatric day clinic also tries to offer help in these cases with consultations by social workers and psychologists. Being free of physical disability seems to be one of the most important factors that influence QoL [3]. Wahrendorf et al. found that there is also a strong association between functional limitations and changes in quality of life; in their study, being free from functional limitations led to a significant increase in QoL [42]. Also in our study, the improvements in mobility and the decrease in fall risk within the overall group were significantly correlated with an improvement in perceived health (Fig. 2). Vellas at al. observed a strong association between fear of falling and a decrease in mobility and QoL in their study [43]. Falls and a fear of falling in turn contribute to restricted activity as a strategy to reduce the perceived risk of subsequent falls [44], which closes the vicious circle. Even depression is related with a higher risk of falls [45]. However, IHHT did not positively affect perceived health more than MTI alone in the present study with geriatric patients. In contrast to mobility, there is no relationship in our study between improvements in exercise tolerance measured with the 6MWT and perceived health assessed with EQ VAS (Fig. 3), which shows that endurance does not influence QoL sufficiently and measurably. It seems that enhancements in mobility, gait and balance directly influence the way in which patients can easily cope with their everyday life and reduces the risk of falls. This, in turn, leads to an increase in the perceived health [42, 43]. Dementia, on the other hand, does not seem to reduce the quality of life for the affected patients; only the affected caregivers show a strong reduction in their own quality of life [46]. In our study, there was no statistical significant correlation between perceived health and cognitive performance (Fig. 3). However, as IHHT leads to an improvement in cognitive performance [10] and as Jing et al. showed that at least caregivers profit from an enhanced cognitive performance of elderly people [46]. IHHT may positively affect elderly patients in several ways [10, 18, 21], but this seems not to be true regarding mobility and perceived health.

### Limitations

First, it is not possible to have the exact same MTI for all patients because of the multi-morbidity of geriatric patients; a special, individual training program has to be created for each person. Second, as muscle weakness is an important risk factor for falls, we did not measure the strength of the lower extremities. Third, we had no control group without intervention to make a valid statement on the effect of MTI on perceived health and fall risk. Fourth, much effort and time of trained supervisors is needed for the individual MTI and IHHT to reach sufficient compliance by elderly people.

## Conclusion

In this study, IHHT added to MTI did not elicit additional improvements in perceived health and mobility compared to MTI alone.

## Data Availability

All relevant data are presented in the manuscript and tables.

## Abbreviations

ACE: Angiotensin Converting Enzyme
ANOVA: Analysis of Variance
AT: Angiotensin
BMI: Body Mass Index
CVD: Cardiovascular Disease
EQ VAS: EuroQol-visual analogue scale
HDL: High Density Lipoprotein
HG: Hypoxic Group
IHHT: Intermittent Hypoxic Hyperoxic Training
IHT: Intermittent Hypoxic Training
LDL: Low Density Lipoprotein
MMSE: Mini-Mental State Examination
MTI: Multimodal Training Intervention
NG: Normoxic Group
QoL: Quality of Life
TMT: Tinetti Mobility Test
TNF: Tumor Necrosis Factor
TUG: Timed-Up-and-Go Test
